# Affective Disorder in Addictology -Salus Hospital

**DOI:** 10.1192/j.eurpsy.2025.978

**Published:** 2025-08-26

**Authors:** T. Pengili, E. Lekli, J. Draci, M. Kambo

**Affiliations:** 1 Psichiatry, Salus Hospital, Tirana, Albania

## Abstract

**Introduction:**

**AFFECTIVE DISORDERS IN ADDICTOLOGY**

In recent years, the number of substance users has increased globally. Referring to a study conducted in Albania by the Youth Risky Behavioral Survey, it results that: 5.4% of young people aged 14-18 have an experience with cannabis, 4% with ecstasy, 1.4% with heroin and 1.6% with cocaine. From the data published by Our World (2019) it results that the number of deaths from drug and alcohol consumption has doubled, especially in the last three decades. From studies it results that 50 thousand individuals in Albania (1.8% of the population are drug users.

From a 3-year study carried out in the Addictology pavilion at Salus Hospital, where a sample of 133 patients with addiction to substances was examined. It turned out that 118 people were men (88.7%) and 15 women (11.3%). Over 50% of patients are in the 18-33 age group and 40% are in the 30-40 age group. Referring to the place of residence, most of the patients are from Tirana and this is influenced by the number of the population (32% of the population of Albania).Education is another important data of this study as it turns out that more than 50% of them have a secondary education and only 1/3 of them have a higher education. Referring to the married/single status, the ratio is respectively 46.97/48.48%.After analyzing the years of abuse,from 1-5 years addiction constitute 29 % of the total,6-10 years constitute 37 % and from 10-20 years constitute 21 %.If we refer to substances, it turns out that cocaine occupies 74% of the championship, followed by Cannabis, 14% and Alcohol, 12%. If we consider the psychiatric pathologies from which this study is referred, it turns out that Affective Disorders make up the largest part with 69% of followed by Psychotic Disorder with 28% of patients. Over 50% of patients manifested symptoms similar to Bipolar Disorder and over 30% of them symptoms of an Uspecified Affective Disorder.

**Objectives:**

Place occupied by mood disorders in addictology

**Methods:**

Quantitative research

**Results:**

Referring to the married/single status,the ratio is respectively 46.97/48.48%.After analyzing the years of abuse, from 1-5 years addiction constitute 29 % of the total,6-10 years constitute 37 % and from 10-20 years constitute 21 %.If we refer to substances,it turns out that cocaine occupies 74% of the championship,followed by Cannabis,14% and Alcohol,12%.If we consider the psychiatric pathologies from which this study is referred,it turns out that Affective Disorders make up the largest part with 69% of followed by Psychotic Disorder with 28% of patients.Over 50% of patients manifested symptoms similar to Bipolar Disorder and over 30% of them symptoms of an Uspecified Affective Disorder

**Conclusions:**

Affective Disorders make up the largest part with 69% of followed by Psychotic Disorder with 28% of patients.Over 50% of patients manifested symptoms similar to Bipolar Disorder and over 30% of them symptoms of an Uspecified Affective Disorder.

**Disclosure of Interest:**

None Declared

